# Trimethylamine-N-Oxide Promotes High-Glucose-Induced Dysfunction and NLRP3 Inflammasome Activation in Retinal Microvascular Endothelial Cells

**DOI:** 10.1155/2023/8224752

**Published:** 2023-02-28

**Authors:** Lidan Xue, Lili Huang, Yajing Tian, Xin Cao, Yu Song

**Affiliations:** Department of Ophthalmology, The Second Affiliated Hospital of Nantong University, Nantong 226000, Jiangsu, China

## Abstract

**Introduction:**

Along with blood glucose levels, diabetic retinopathy (DR) development also involves endogenous risk factors, such as trimethylamine-N-oxide (TMAO), a product of intestinal flora metabolic disorder, which exacerbates diabetic microvascular complications. However, the effect of TMAO on retinal cells under high-glucose conditions remains unclear. Therefore, this study examined the effects of TMAO on high-glucose-induced retinal dysfunction in the context of NLRP3 inflammasome activation, which is involved in DR.

**Materials and Methods:**

TMAO was assessed in the serum and aqueous humor of patients using ELISA. Human retinal microvascular endothelial cells (HRMECs) were treated for 72 h as follows: NG (normal glucose, D-glucose 5.5 mM), NG + TMAO (5 *μ*M), HG (high glucose, D-glucose 30 mM), and HG + TMAO (5 *μ*M). The CCK8 assay was then used to assess cell proliferation; wound healing, cell migration, and tube formation assays were used to verify changes in cell phenotype. ZO-1 expression was determined using immunofluorescence and western blotting. Reactive oxygen species (ROS) formation was assessed using DCFH-DA. NLRP3 inflammasome complex activation was determined using a western blot.

**Results:**

The serum and aqueous humor from patients with PDR contained higher levels of TMAO compared to patients with nontype 2 diabetes (Control), non-DR (NDR), and non-PDR (NPDR). TMAO showed significant acceleration of high-glucose-induced cell proliferation, wound healing, cell migration, and tube formation. ZO-1 expression decreased remarkably with the combined action of TMAO and a high glucose compared to either treatment alone. TMAO also promoted high-glucose-activated NLRP3 inflammasome complex.

**Conclusion:**

The combination of TMAO and high-glucose results in increased levels of ROS and NLRP3 inflammasome complex activation in HRMECs, leading to exacerbated retinal dysfunction and barrier failure. Thus, TMAO can accelerate PDR occurrence and development, thus indicating the need for early fundus monitoring in diabetic patients with intestinal flora disorders.

## 1. Introduction

Diabetic retinopathy (DR) is a common microvascular disorder complicated by diabetes and is now the leading cause of blindness and visual impairment among the working-age population [1]. With DR progression, it can be divided into nonproliferative DR (NPDR) and proliferative DR (PDR). NPDR usually demonstrates microaneurysms, intraretinal microvascular abnormalities (IRMA), increased vessel permeability, and hard and soft exudations. Blood flow changes, loss of pericytes, broken tight junctions of endothelial cells, and pachynsis of capillaries in the endothelial basement membrane form the pathological basis of NPDR [2]. The core process of PDR is neovascularization, with gradual ischemia and hypoxia [3]. Oxidative stress and inflammatory reactions play significant roles in pathological processes throughout the development of diabetic retinal vasculopathy [4]. Anti-VEGF targeting has been widely used as an effective intervention in patients with PDR and diabetic macular edema. Studies on the Joslin 50-year Medalist cohort, comprising patients suffering from insulin-dependent diabetes for 50 years or more, have indicated no significant correlation between the severity of diabetic retinopathy and the level of blood glucose control [5]. This suggests that endogenous risk factors and protective factors other than glucose levels may be involved in the occurrence and development of DR.

Trimethylamine N-oxide (TMAO) is an amine oxide that can be induced by gut dysbiosis [6]. Choline, betaine, and carnitine, which originate from animal dietary components, are converted into trimethylamine (TMA) under the influence of the gut microbiota [7]. TMA is transported into the liver through the portal circulation, where it is oxidized to TMAO by flavin-containing monooxygenase 3 (FMO3) [8]. Several studies have shown that most cardiovascular diseases (CVD) and renal diseases are closely related to TMAO [9]. A recent review indicated that gut dysbiosis and the development of type 2 diabetes (T2DM) as well as the related retinal, neurological, and renal microvascular complications went hand in hand [10]. TMAO might play a key role in diabetic cardiomyopathy (DCM) through the pathways of inflammation, connexin remodeling, the increase of calcium ions (Ca^2+^), myocardial fibrosis, and so on [11, 12]. In addition, TMAO could activate renal inflammation, oxidative stress, fibrosis, and endothelial dysfunction in the pathogenesis of diabetic kidney disease (DKD) [13, 14]. A clinical study demonstrated that elevated TMAO concentrations in plasma were associated with increased incidence and severity of DR in T2DM [15]. However, the specific mechanism of TMAO as a risk factor for DR and its effect on retinal cells under the action of high glucose remain unclear.

The NLRP3 inflammasome has been found to be upregulated in the retinal proliferative membranes of PDR patients as well as in *in vitro* and *in vivo* DR models [16]. Once inflammatory stimuli are sensed, activated NLRP3 binds the adaptor protein ASC, which contains a pyrin domain (PYD) and caspase recruitment domain (CARD), to recruit and cleave caspase-1. This triggers the downstream reaction, resulting in interleukin-1*β* (IL-1*β*) release [17]. Considering the role of TMAO as a risk element in DR, it is important to examine whether TMAO can enhance NLRP3 inflammasome activation in DR. In hyperglycemia, dysfunction and activation of the NLRP3 inflammasome are found in retinal microvascular endothelial cells (RMECs) [18].

Therefore, in this study, we examined the mechanism by which TMAO is involved in the dysfunction and activation of NLRP3 inflammasomes in RMECs under a high-glucose environment.

## 2. Materials and Methods

### 2.1. Serum and Aqueous Humor Sampling and Quantitative TMAO Analyses

Participants with or without type 2 diabetes who had cataract extraction and participants with PDR and type 2 diabetes who underwent vitrectomy at the Second Affiliated Hospital of Nantong University were recruited. This study followed the guidelines of the Declaration of Helsinki. Sampling was carried out with the informed consent of patients and approval from the hospital ethics committee. The samples were stored at −80°C. The concentrations of TMAO in serum and aqueous humor samples were determined using a human TMAO ELISA kit (JINGME, JiangSu, China), according to the manufacturer's protocol; the results were determined at 450 nm using a microplate reader (BIOTEK, USA). The TMAO concentrations were calculated based on standard concentrations and expressed as ppm (mg/L).

### 2.2. Cell Culture and Treatments

A human retinal microvascular endothelial cell line (HRMEC) was purchased from the BeNa Culture Collection (Beina Chuanglian Biotechnology Institute, Beijing, China). Cells (passages 3–6) were cultured in D-glucose (5.5 mM)-containing DMEM (HyClone, UTAH, USA) containing 5% fetal bovine serum (FBS) at 37°C (95% air, 5% CO_2_). When the cells reached confluence after seeding, the medium was replaced as follows: NG (Normal Glucose, D-glucose 5.5 mM), OSM (Osmotic, Mannitol 24.5 mM, and D-glucose 5.5 mM), NG (Normal Glucose, D-glucose 5.5 mM) + TMAO (5 *μ*M, Sigma, Japan), HG (High Glucose, D-glucose 30 mM), and HG (High Glucose, D-glucose 30 mM) + TMAO (5 *μ*M) in DMEM with 5% FBS for 72 h before the experiments.

### 2.3. CCK8 Assay

HRMECs were seeded into 96-wells plates (5000 cells/well) in D-glucose (5.5 mM) DMEM medium with 5% FBS for 24 hours, and then treated with D-glucose (5.5 mM) + TMAO (0 *μ*M), D-glucose (30 mM)) + TMAO (0 *μ*M), D-glucose (30 mM) + TMAO (1.25 *μ*M), D-glucose (30 mM) + TMAO (2.5 *μ*M), D-glucose (30 mM) + TMAO (5 *μ*M), D-glucose (30 mM) + TMAO (10 *μ*M), D-glucose (30 mM) + TMAO (20 *μ*M), D-glucose (30 mM) + TMAO (50 *μ*M), and D-glucose (30 mM) + TMAO (100 *μ*M). At the indicated time points (24 h, 48 h, and 72 h), CCK-8 solution (10 *μ*L/well, Proteintech, IL, USA) was added to the wells and incubated at 37°C for 2 h. Absorbance was detected at a wavelength of 450 nm using a microplate reader.

### 2.4. ROS Assessment

HRMECs were seeded into a 6-well plate and treated as needed. Cells were then loaded with DCFH-DA (10 *μ*M, Beyotime Biotech, Shanghai, China) in serum-free medium and incubated for 20 minutes at 37°C. After washing 3 times with phosphate buffered saline, the images were captured under an inverted fluorescence microscope (ECLIPSE Ti2, NIKON, Japan). Fluorescent intensity was analyzed using Image J software.

### 2.5. Cell Migration and Healing Assays

For migration assays, migration chambers were placed in a 24-well plate and pretreated HRMECs were seeded into the upper chamber. Then, 4% paraformaldehyde was used to fix the cells, and they were incubated for 12 h. Cells were then stained with crystal violet and counted under a microscope as follows: For wound healing assays, HRMECs were incubated on a 6-well plate until confluence and then scratched with sterile 200 *μ*L pipette tips. The wells were then viewed under an inverted microscope (TS2, NIKON, Japan) and the wound healing percentage was calculated after treatment for 24 h, 48 h, and 72 h. The scratch areas were calculated by Image J software, and then the wound healing areas were obtained by calculating the *D*-value between the scratch areas of 24, 48, and 72 hours and the initial scratch area of this group. The wound healing percentages were the ratios of the wound healing areas to the initial scratch area of the group.

### 2.6. Tube Formation Assay

Matrigel (356234, Corning, NY, USA) was used to coat a 96-well plate (50 *μ*L/L), which was subsequently incubated at 37°C for 45 minutes to solidify. Pretreated cell suspensions were then seeded on the solidified Matrigel (20,000 cells/well). After incubation at 37°C for 6 h, the tube formation of HRMECs was observed using an inverted microscope. The tube length was analyzed using Image J software.

### 2.7. Immunofluorescence Analysis for ZO-1

After treatment, HRMECs were fixed using 4% paraformaldehyde for 15 minutes and then blocked with 1% BSA in PBS for 2 h at 37°C. The cells were then incubated overnight at 4°C with anti-ZO-1 (1 : 400; Cell Signaling), followed by incubation with red-labeled antirabbit secondary antibody(1 : 500, Invitrogen, CA, USA) for 2 h at room temperature. The cells were sealed with antifade mounting medium containing DAPI (Beyotime Biotech, Shanghai, China) and scanned under a fluorescence microscope (ECLIPSE NI-E, NIKON, Japan). Fluorescent intensity was analyzed using Image J software.

### 2.8. Western Blot

Treated cells were lysed in RIPA containing protease inhibitor (PMSF, 1 : 100) on ice for 30 minutes. The cell lysate was then centrifuged at 12000 rpm at 4°C for 15 min. The supernatant was then transferred to new centrifuge tubes. The protein concentrations were determined using a BCA kit (Beyotime Biotech, Shanghai, China); equal amounts (40 *μ*g) of protein samples were separated using SDS-PAGE and then transferred to polyvinylidene fluoride (PVDF) membranes. The membranes were blocked with 5% skimmed milk powder in TBST for 2 h. The membranes were probed overnight at 4°C with anti-NLPR3 (1 : 1000, Abcam, UK), anti-ASC (1 : 1000, Proteintech, IL, USA), anti-Caspase1 (1 : 1000, Proteintech, IL, USA), and anti-IL-1*β* antibodies (1 : 1000, Proteintech, IL, USA). Further, the membranes were incubated with HRP-conjugated secondary antibodies(1 : 2000, Proteintech, IL, USA) for 2 h at room temperature. The antibody binding was detected using the enhanced chemiluminescence (ECL) detection kit (Beyotime Biotech, Shanghai, China).

### 2.9. Statistical Analysis

Statistical analysis was performed using GraphPad Prism version 8.0 and SPSS 23. The data are presented as mean ± standard deviation (SD). A one-way ANOVA followed by Tukey's post hoc test was used to examine the significant differences between and within different groups. The Kruskal−Wallis test was used for nonnormally distributed data. To compare the differences in clinical characteristics between the four groups, we used the Wilcoxon and McNemar tests as appropriate. The level of statistical significance was set at *P* < 0.05.

## 3. Results

### 3.1. TMAO Showed Higher Levels in Proliferative Diabetic Retinopathy (PDR)

**Figure 1:**
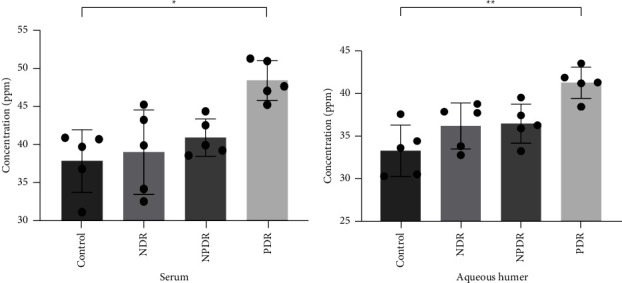
TMAO is present at higher concentrations in patients with proliferative diabetic retinopathy. Mean TMAO concentrations in serum (a) and aqueous humor. (b) CT: control group; NDR: non-DR group; NPDR: non-PDR group; PDR: PDR group. ^*∗*^*Pvs.* PDR; ^*∗*^indicates *P* < 0.05; *P* values were determined using Kruskal−Wallis test as appropriate.

We assessed the concentration of TMAO in the samples from four groups of participating patients: control group (CT): cataract without diabetes; non-DR group (NDR): cataract with diabetes but not diabetic retinopathy; non-PDR group (NPDR): cataract with nonproliferative diabetic retinopathy; and PDR group (PDR): patients with proliferative diabetic retinopathy who underwent vitrectomy. Each group contained 5 patients, and the clinical characteristics of the participants were shown in Table 1. No significant differences in age, gender, or diabetes duration among the four groups are displayed in Table 1. Compared with the other three groups, the PDR group showed significantly higher expression of TMAO in both serum (Figure 1(a)) and aqueous humor samples (Figure 1(b)). However, there was no statistically significant difference among the other three groups. This suggests that TMAO may accelerate the progression of diabetic retinopathy and aggravate its condition.

### 3.2. TMAO Promotes Cell Proliferation Induced by High Glucose in Human Retinal Microvascular Endothelial Cells

**Figure 2:**
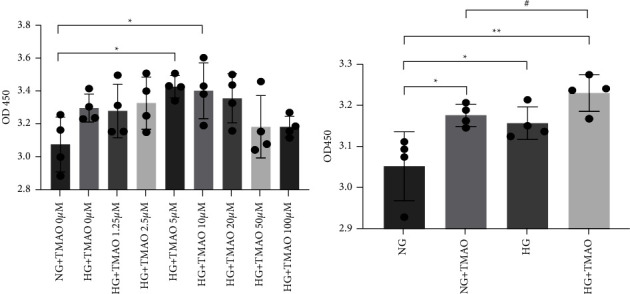
TMAO promotes cell proliferation induced by high glucose in human retinal microvascular endothelial cells. HRMECs were treated with NG (normal glucose, D-glucose 5.5 mM) + TMAO (0 *μ*M) and HG (high glucose, D-glucose 30 mM) + TMAO (0, 1.25, 2.5, 5, 10, 20, 50, 100 *μ*M) for 72 h. (a) OD450 values were read after 2 h of adding CCK8 solution. ^*∗*^*P* < 0.05*vs.* NG + TMAO (0 *μ*M), *n* = 4. HRMECs were treated with NG (D-glucose, 5.5 mM), NG + TMAO (5 *μ*M), HG (D-glucose, 30 mM), and HG + TMAO (5 *μ*M) for 72 h. (b) OD450 values were read after 2 h of adding CCK8 solution. ^*∗*^*P* < 0.05*vs.* NG; ^#^*P* < 0.05*vs*. NG + TMAO, *n* = 4.

To investigate the effect of TMAO in HRMECs treated with high glucose (HG, D-glucose 30 mM), the cells were treated with NG (normal glucose, D-glucose 5.5 mM) + TMAO (0 *μ*M) or HG + TMAO (0, 1.25, 2.5, 5, 10, 20, 50, or 100 *μ*M) for 72 hours. The CCK8 assay was then used to determine the cell proliferation based on the OD 450 value. As shown in Figure 2(a), TMAO at 2.5, 5, 10, and 20 *μ*M could promote the cell proliferation induced by HG, and only HG + TMAO (5 or 10 *μ*M) showed statistically significant differences compared with NG + TMAO (0 *μ*M) at 72 h; cell proliferation was also better increased with TMAO 5 *μ*M than 10 *μ*M. Therefore, 5 *μ*M TMAO was used in the subsequent experiments. We continued to compare the cell proliferation in the four different groups; as shown in Figure 2(b), the cell proliferation in NG + TMAO (5 *μ*M), HG, and HG + TMAO (5 *μ*M) was significantly increased compared with that in NG at 72 h. Moreover, the combination of TMAO and HG could accelerate cell proliferation compared with that induced by HG or TMAO alone. Overall, high-glucose-induced proliferation of HRMECs can be increased by TMAO.

### 3.3. TMAO Enhances High-Glucose-Induced Wound Healing, Migration, and Vascular Tube Formation of Human Retinal Microvascular Endothelial Cells

**Figure 3:**
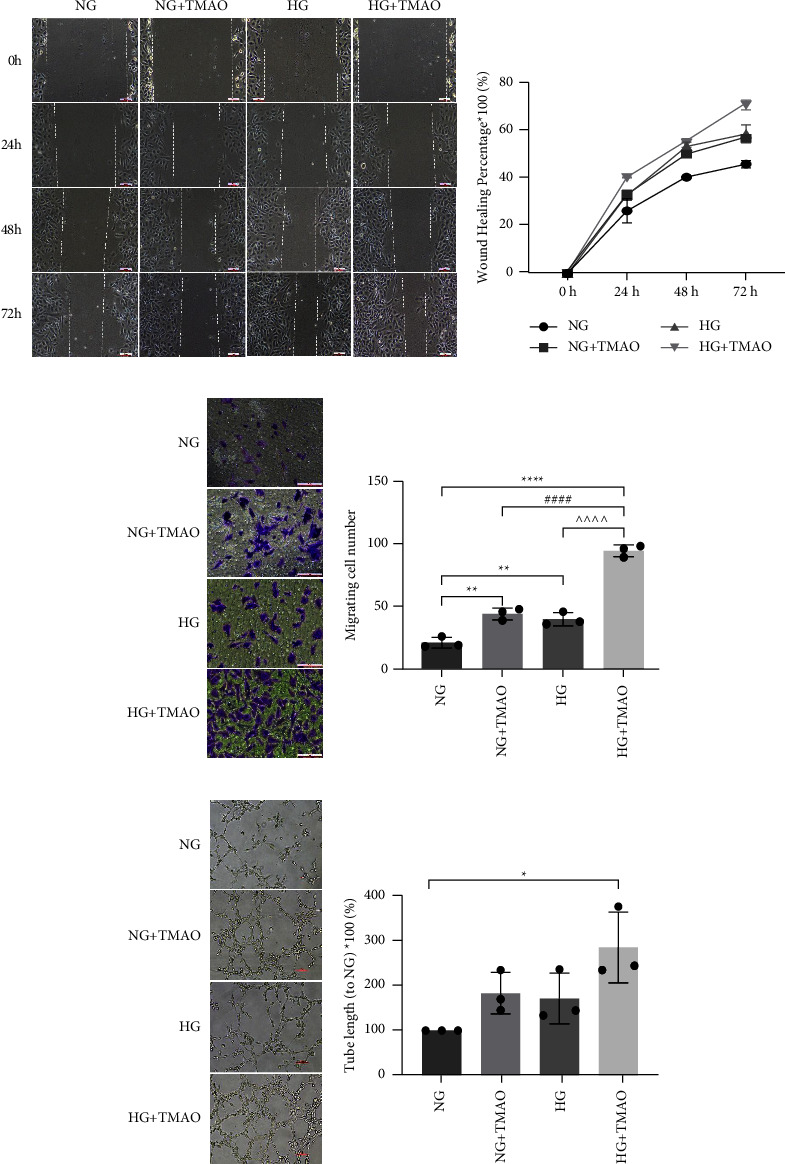
TMAO enhances high-glucose induced wound healing, migration and vascular tube formation of human retinal microvascular endothelial cells. In the wound healing assay, TMAO accelerated the healing process induced by high glucose as shown in the images. (a) Scale, 100 *μ*m. Quantitative analysis of wound healing percentage. (b) Cell migration was determined using a transwell assay after cells were treated for 72 h. (c) Scale, 100 *μ*m. Number of migrated cells. (d) After different treatments for 72 h, HRMECs were seeded into wells coated with matrigel for 6 h, and tube formation was observed. (e) Scale, 50 *μ*m. Tube lengths were compared to NG. (f) ^*∗*^*P* < 0.05*vs.* NG; ^^^*P* < 0.05*vs.* HG; ^#^*P* < 0.05*vs.* NG + TMAO, *n* = 3.

In our previous research, we detected that osmotic pressure had no effect on the migration and tube formation of HRMECs [19]. Thus, we did not conduct osmotic controls in this experiment. Both TMAO and high glucose can individually promote the wound healing of HRMECs, but when combined, the healing process could be accelerated further (Figures 3(a) and 3(b)). To assess cell migration, we used the Transwell chamber and found that the number of migrating cells was significantly increased when HRMECs were cultured with HG and TMAO together, compared with the individual HG and TMAO treatments (Figures 3(c) and 3(d)). Tube formation was also more striking after costimulation with TMAO and high glucose (Figures 3(e) and 3(f)). These results indicate that overexpression of TMAO accelerates neovascularization under high glucose conditions.

### 3.4. TMAO Enhances High-Glucose-Induced Degradation of the Tight Junction Protein ZO-1 in HRMECs

**Figure 4:**
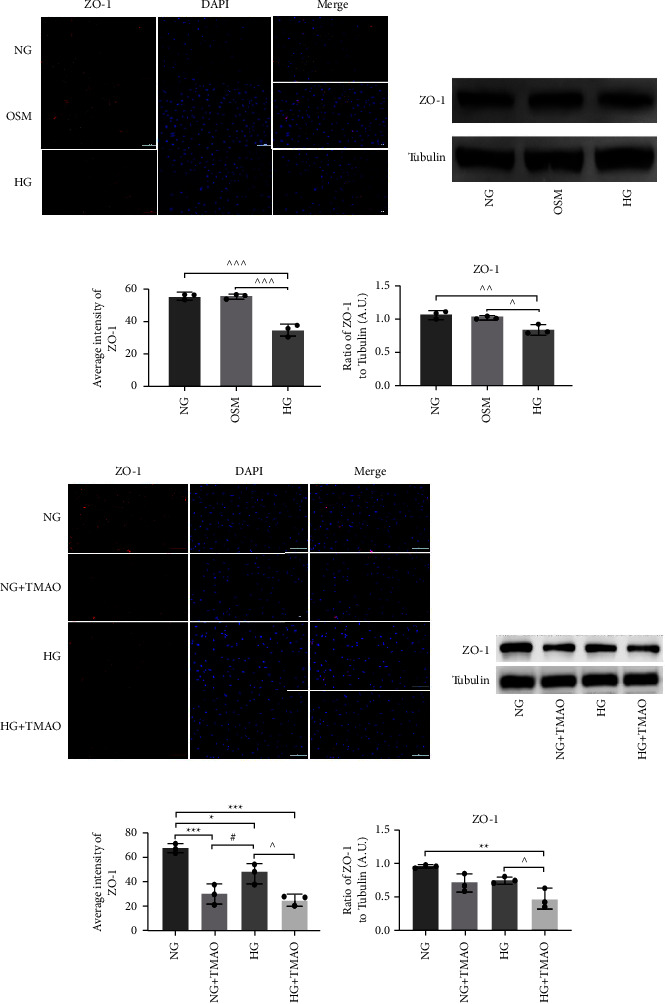
TMAO enhances the high-glucose-induced damage to tight junction protein ZO-1 in HRMECs. After 72 h exposure to NG (D-glucose 5.5 mM), OSM (mannitol 24.5 mM and D-glucose 5.5 mM), and HG (D-glucose 30 mM), the fluorescence and protein expression of ZO-1 were displayed (a‐d). Fluorescence images showing ZO-1 expression in HRMECs after treatment with NG, NG + TMAO, HG, and HG + TMAO for 72 h (e, g). Scale, 100 *μ*m. Western blot analysis of ZO-1 expression (f, h). ^∗^*P*<0.05 vs. NG, ^^^ *P*<0.05 vs. HG, #*P*<0.05 vs. NG + TMAO, n = 3.

We then examined ZO-1 expression by HRMECs after different treatments for 72 h. As results illustrated, OSM (mannitol 24.5 mM and D-glucose 5.5 mM) exposure produced no effect on the fluorescence and protein expression of ZO-1 compared to NG (D-glucose 5.5 mM) and HG (D-glucose 30 mM) (Figures 4(a)–4(d)). Red fluorescence intensity of ZO-1 decreased significantly upon combined stimulation with TMAO and high glucose compared to that with either treatment alone (Figures 4(e) and 4(g)). Western blot analysis further confirmed a more serious decrease in ZO-1 protein levels with the joint action of TMAO and high glucose (Figures 4(f) and 4(h)). ZO-1 protein is a component of the intercellular tight junctions. When tight junctions are disintegrated, ZO-1 expression decreases. Our results thus indicate that TMAO may accelerate the destruction of tight junctions induced by high glucose in HRMECs. This may suggest that endothelial integrity could be decreased and high glucose-induced vascular leakage could be aggravated by TMAO.

### 3.5. TMAO Can Increase High Glucose-Induced ROS

**Figure 5:**
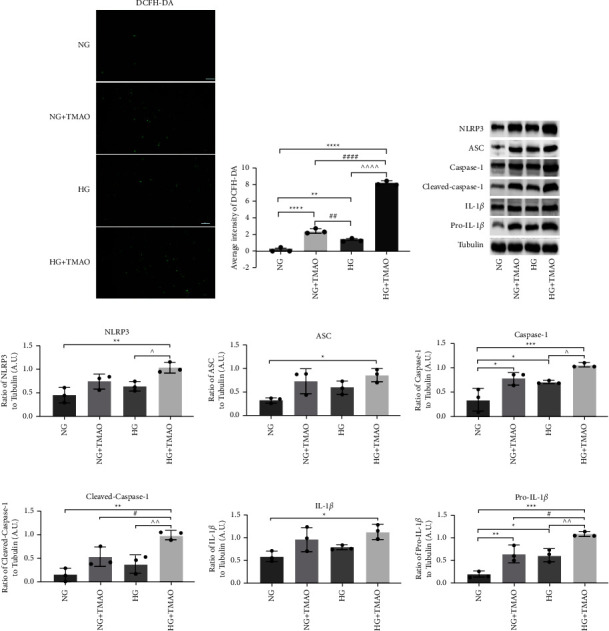
TMAO enhances high-glucose-induced ROS and NLRP3 inflammasome signaling activation. Reactive oxygen species (ROS) levels in HRMECs treated for 72 h, detected using DCFH-DA. Green fluorescence represents the positive ROS expression. (a) Scale, 50 *μ*m. Average intensity of green fluorescence. (b) After treatment with NG, NG + TMAO, HG, HG + TMAO for 72 h, NLRP3 inflammasome proteins were measured using western blot. (c) NLRP3. (d) ASC. (e) Caspase-1. (f) Cleaved-caspase-1. (g) IL-1. (h) Pro-IL-1*β*. (i) Expression ratios compared to tubulin. ^*∗*^*P* < 0.05*vs.* NG; ^^^*P* < 0.05*vs.* HG; ^#^*P* < 0.05*vs.* NG + TMAO, *n* = 3.

The intracellular ROS levels after 72 h of different stimulations were found to be significantly upregulated by TMAO and high glucose individually, but were further increased by the combination of TMAO and high glucose (Figures 5(a) and 5(b)). Previous studies have demonstrated that upregulation of oxidative stress can lead to inflammation in the DR. These results indicate that TMAO can enhance high glucose-induced ROS, suggesting that the inflammation induced by high glucose can also be exacerbated by exposure to TMAO.

### 3.6. TMAO Can Enhance High-Glucose-Induced NLRP3 Inflammasome Signaling Activation

High glucose can induce inflammasome activation, which plays an important role in the occurrence and development of DR [20]. In this study, we investigated whether TMAO could enhance the NLRP3 inflammasome activation induced by high-glucose (Figure 5(c)). The protein expression levels of NLRP3 (Figure 5(d)), ASC (Figure 5(e)), Caspase-1 (Figure 5(f)), cleaved-Caspase-1 (Figure 5(g)), IL-1 (Figure 5(h)), and Pro-IL-1*β* (Figure 5(i)) were found to be increased by the combined treatment with TMAO and high glucose compared to that induced by either TMAO or high glucose alone. Thus, TMAO may accelerate the occurrence of DR by increasing the inflammasome activation induced by high glucose.

## 4. Discussion

We found that the TMAO content in serum and aqueous humor were both much higher in the PDR group than in the CT, NDR, and NPDR groups. Our study aimed to confirm whether TMAO can accelerate high-glucose-induced dysfunction and NLRP3 inflammasome activation in HRMECs to accelerate the pathogenesis of DR. We found that TMAO can enhance the proliferation, wound healing, migration, tube formation, and degradation of intercellular tight junctions induced by high glucose in HRMECs. Thus, TMAO could accelerate high glucose-caused neovascularization and vascular leakage. Interestingly, we found that a 5 *μ*M concentration of TMAO combined with high-glucose guided greater cell proliferation than 10 *μ*M as well as higher concentrations. On the one hand, it was reported that TMAO could activate oxidative stress to induce pyroptosis in the vascular endothelial cells [21] or apoptosis in renal cells [22]. On the other hand, apoptosis in the pancreatic acinar cells has been investigated and could occur after treatment with TMAO via ER stress [23]. So, with the increase in TMAO concentration, we guess that pyroptosis or apoptosis may occur in the HRMECs. In that case, 5 *μ*M concentration of TMAO could induce greater cell proliferation than higher concentration of TMAO in HRMECs. We demonstrated that TMAO can enhance the high-glucose-induced occurrence of oxidative stress and NLRP3 inflammasome complex activation. Pathological neovascularization is known to play a key role in the development of DR [24]. Oxidative stress and NLRP3 inflammasome complex activation, including the subsequent chronic inflammation, are also crucial pathogenic processes in DR [25, 26]. Gut dysbiosis has been indicated to be associated with the progression of diabetic microvascular complications, including DR, and TMAO overexpression can be induced by gut dysbiosis [10]. Based on these findings, TMAO overexpression in the retina can accelerate the occurrence and development of DR. The plasma levels of TMAO are reported to be associated with the incidence rate and severity of DR [15]. Our experimental results were consistent with these clinical findings. However, we did not further investigate additional signaling pathways for the combined action of TMAO and high glucose in HRMECs; therefore, we attempted to conjecture the related mechanisms based on previous studies on DR and another diabetic vascular complication associated with TMAO.

The PKC (protein kinase C) pathway plays a key role in the oxidative stress of DR [27]. Hyperglycemia increases the synthesis of diacylglycerol (DAG) through glycolysis, which is an agonist of PKC [28]. PKC can enhance active NADPH oxidase and regulate the assembly and activation of NOX 2 and NOX 4 isoforms, which can further increase the level of oxidative stress in retinal cells [29]. PKC activity can also increase NF-*κ*B phosphorylation, which can increase inflammation, activation of the NLRP3 inflammasome, and loss of ZO-1 and Claudin-1, thus damaging the blood –retinal barrier [30–32]. TMAO has also been found to activate PKC in human umbilical vein endothelial cells (HUVECs) to induce monocyte adhesion [33]. TMAO may thus enhance high-glucose-induced oxidative stress through the PKC pathway.

The NLRP3 inflammasome can also be activated by several other pathways, including toll-like-receptor-4 (TLR4) [34], p38 [35], Nrf2 [36, 37], ROS [38], PKR [39], and prostaglandin E [40, 41], in retinal cells. In cardiovascular disease, TMAO has been indicated to activate the NLRP3 inflammasome through ROS induction [41]. Therefore, we consider that TMAO can promote ROS to further accelerate high-glucose-induced activation of the NLRP3 inflammasome.

Based on our findings, TMAO can accelerate the high-glucose-induced destruction of tight junctions; however, TMAO itself can activate the NLRP3 inflammasome and induce dysfunction in HRMECs. It is widely reported that TMAO can increase the risk of cardiovascular disease [42, 43]. Disorders of the gut microbial ecosystem are reported to increase the risk of cardiovascular disease (CVD), atherosclerosis, diabetes, and stroke [44, 45]. As products of intestinal dysbacteriosis, TMAO may thus be a risk factor for ocular fundus diseases; thus, regular examination of the ocular fundus is necessary, especially in patients with diabetes.

## 5. Conclusion

In conclusion, our study confirmed higher levels of TMAO in patients with PDR. We further demonstrated that TMAO can promote NLRP3 inflammasome activation and HRMEC dysfunction induced by high glucose. TMAO overexpression may thus accelerate the course of DR and loss of vision. Therefore, fundus monitoring needs to be conducted as early as possible in diabetic patients with intestinal flora disorders. But this study also has some limitations, such as the fact that no animal experiments were conducted to further verify this study and the number of clinical participants was small. In the future, we will recruit more participants in our research and conduct more experiments to explore the mechanism of TMAO with DR.

## Figures and Tables

**Table 1:** Clinical characteristics of the participants.

Variables	Controls	NDR	NPDR	PDR	*χ* ^2^ value	*P* value
*n*	5	5	5	5		
Age (year)	65.2 ± 7.46	67.2 ± 7.69	60.6 ± 7.27	57.4 ± 3.50	6.187	0.103
Female (%)	60	40	60	40		0.861
Diabetes duration (year)	NS	7.0 ± 3.46	8.20 ± 1.79	10.60 ± 0.89	4.111	0.128

*P* values were determined using Wilcoxon test and McNemar test as appropriate.

## Data Availability

All data generated or analyzed during this study are included in this article. Further enquiries can be directed to the corresponding author.
